# Circular RNA circPPP6R3 upregulates CD44 to promote the progression of clear cell renal cell carcinoma via sponging miR-1238-3p

**DOI:** 10.1038/s41419-021-04462-5

**Published:** 2021-12-21

**Authors:** Zhiliang Chen, Zaosong Zheng, Yingwei Xie, Qiyu Zhong, Wentai Shangguan, Yishan Zhang, Dingjun Zhu, Wenlian Xie

**Affiliations:** 1 Department of Urology, Sun Yat-sen Memorial Hospital, Sun Yat-sen University, Guangzhou, 510120 China; 2 Guangdong Provincial Key Laboratory of Malignant Tumor Epigenetics and Gene Regulation, Sun Yat-sen Memorial Hospital, Sun Yat-sen University, Guangzhou, 510120 China; 3 Department of Urology, Nanfang Hospital, Southern Medical University, Guangzhou, 510515 China

**Keywords:** Tumour biomarkers, Renal cell carcinoma, Epithelial-mesenchymal transition, Non-coding RNAs

## Abstract

Circular RNAs (circRNAs) are a type of covalently closed circular-formed RNAs and play crucial roles in the oncogenesis and progression of various human cancers. Here we identified a novel circRNA, circPPP6R3, to be highly expressed both in clear cell renal cell carcinoma (ccRCC) tissues and cell lines based on analyzing high-throughput sequencing data and qRT-PCR analysis. Highly expressed circPPP6R3 was positively correlated with higher histological grade, T stage, and M stage as well as advanced clinical stage of ccRCC patients. Functionally, knockdown of circPPP6R3 attenuated the proliferation, migration, and invasion of ccRCC cells whereas overexpression had the reverse effects. Mechanistically, the biotin-labeled pull-down assay and dual-luciferase reporter assay revealed that circPPP6R3 directly interacted with miR-1238-3p. miR-1238-3p inhibitors had a rescue effect on the proliferative and metastatic capacities by knockdown of circPPP6R3. Moreover, RNA-sequencing analysis and dual-luciferase reporter assay indicated that circPPP6R3 upregulated CD44, a cell-surface glycoprotein contributed to the cell adhesion and metastasis, via sponging to miR-1238-3p. Further investigation revealed that MMP9 and Vimentin were regulated by CD44 in ccRCC. Our study thus provided evidence that the regulatory network involving circPPP6R3/miR-1238-3p/CD44 axis might provide promising biomarkers as well as a therapeutic approach for ccRCC.

## Introduction

Renal cell carcinoma (RCC) is one of the most common malignant tumors of the urinary system. According to the 2019 European Association of Urology guidelines, RCC comprises ~3% of all cancers. In 2018, the number of new RCC cases in European countries was 99,200, an annual increase of 2% during the past 20 years, and the number of deaths related to RCC reached 39,100 [1]. RCC originates from renal tubular epithelial cells and represents ~85–90% of renal malignancies [2], and clear cell RCC (ccRCC) is the most common histological type of RCC, accounting for ~75% of all histological types [3]. RCC is insensitive to chemotherapy or radiotherapy, which means surgery is the only curative treatment to treat localized and locally advanced RCC. However, nearly 30% of RCC patients developed recurrence or metastasis after surgical management [4], beyond that, 20–25% of RCC patients have already developed distant metastases when diagnosed at the first time [5]. Although targeted therapy has been applied to improve the patient’s survival to a certain extent [6], the therapeutic effect of which varies from person to person [7]. Most metastatic RCC patients occurred progression eventually and the median survival time of metastatic RCC is only 14.8 months [8]. Therefore, it is of great importance to investigate the profound molecular mechanisms underlying the metastasis of RCC and to find new biomarkers and therapeutic targets facilitating a better diagnosis and treatment of RCC.

Recently, accumulated evidence has revealed that circular RNAs (circRNAs), a subclass of non-coding RNAs, contribute to the oncogenesis and progression in various human cancers. circRNAs are a type of covalently closed circular-formed RNAs, without 5' caps or 3' poly-A tails [9]. They are characterized by conserved stability and spatiotemporal specificity [10, 11]. Previous studies have demonstrated that circRNAs could function through sponging to miRNAs, interacting with proteins [12], regulating gene transcription [13], or translating to peptides and proteins [14]. Among these, circRNAs acting as competing endogenous RNAs (ceRNAs) have been widely reported [15]. For instance, circTP63 could competitively sponge to miR-873-3p to enhance the expression of FOXM1 and promote the proliferation of lung squamous cell carcinoma [16]. Exosomal circSHKBP1 functions as a ceRNA to regulate the miR-582-3p/HUR/VEGF axis as well as represses the degradation of HSP90, which leads to the promotion of gastric cancer progression [17]. Although several RCC-related circRNAs have already been identified [18–20], the vital roles of circRNAs in the progression of RCC still need to be elucidated comprehensively.

In this study, we investigated the dysregulated circRNAs by conducting bioinformatics analysis with four paired ccRCC circRNA chip analysis from the GEO database [21] as well as the differentially expressed circRNAs between ccRCC cell line and renal tubular epithelial cell line in Cancer-Specific circRNA network database [22]. Then, a significantly upregulated circRNA, circPPP6R3, was identified to be positively correlated with high pathological grade, clinical stage, T stage, and M stage of ccRCC and might play an oncogenic role in the progression of ccRCC via miR-1238-3p/CD44 axis. Thus, our results indicated that circPPP6R3 might serve as a promising biomarker for diagnosis and potential therapeutic targets in ccRCC.

## Results

### The identification and characteristics of circPPP6R3 in ccRCC

**Fig. 1:**
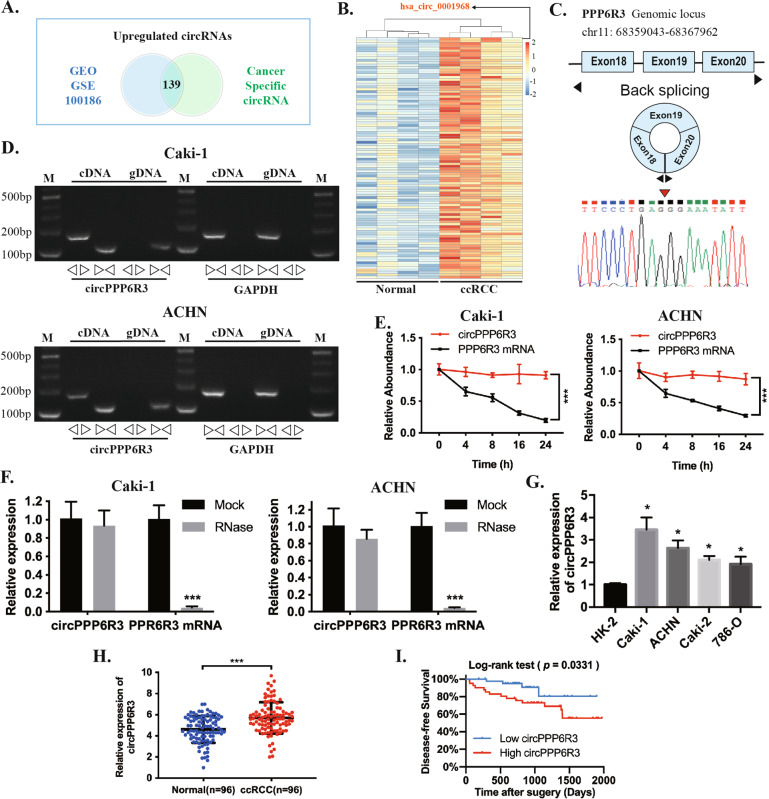
circRNA expression profiles in ccRCC and characteristics of circPPP6R3. **A** The upregulated circRNAs were screened by two online databases, GSE100186 comprised of four paired ccRCC tissues and normal tissues in the GEO database as well as the Cancer-Specific CircRNA database. **B** The cluster heatmap was conducted to present the top 100 upregulated circRNAs of this intersection in the GSE100186 dataset. **C** circPPP6R3 was produced by back-spliced of the exon 18, 19, and 20 of the PPP6R3 gene identified by sanger sequencing. **D** The PCR products of circPPP6R3 and its linear gene PPP6R3 mRNA from the cDNA and gDNA of ccRCC cells were tested by Northern Blotting, GAPDH was used as a control. **E** Actinomycin D was applied to detect the stability of circPPP6R3 and PPP6R3 mRNA. **F** The expression of circPPP6R3 and PPP6R3 mRNA were validated by qRT-PCR under the digestion of RNase R in ccRCC cells. **G** The expression of circPPP6R3 was determined by qRT-PCR in ccRCC cell lines and the renal tubular epithelial cell line, HK-2. **H** qRT-PCR analysis of circPPP6R3 in 96 paired of ccRCC tissues and adjacent normal tissues. **I** The Kaplan–Meier analysis indicated that the ccRCC patients with high expression of circPPP6R3 had unfavorable disease-free survival rates compared with low circPPP6R3 expression.

We first co-analyzed the results from GEO, GSE100186 dataset comprising of four paired ccRCC tissues and the adjacent normal renal tissues, along with the results of the differentially expressed circRNAs between the ccRCC cell line Caki-2 and HK-2, the renal tubular epithelial cell line (Fig. 1A). The intersection revealed that 139 circRNAs (fold change ≥2.0 and *p* < 0.05) were upregulated both in ccRCC tissues and cell lines, a clustered heatmap was employed to show the top 100 upregulated circRNAs (Fig. 1B and Supplementary File 1). Among the upregulated circRNAs in such intersection, the circRNA circPPP6R3 (identified as hsa_circ_0001968 in circBase database) was the most remarkably upregulated circRNAs in ccRCC tissues with a fold change of 29.7. circPPP6R3 was back-spliced of the exon 18, 19, and 20 originated from the PPP6R3 gene (Genomic locus—chr11: 68359043-68367962) (Fig. 1C). Then, we designed the convergent primers to amplify the linear form of PPP6R3 mRNA and the divergent primers to amplify the back-spliced form of circPPP6R3, the genomic DNA (gDNA) and complementary DNA (cDNA) were employed as templates. The nucleic acid electrophoresis implied that divergent primers could amplify circPPP6R3 only in cDNA but not in gDNA, and the back-spliced junction was verified by sanger sequencing (Fig. 1D). As reported in previous studies, the circular structure of circRNAs endows them with excellent stability. Therefore, we adopted actinomycin D to inhibit the transcription in ccRCC cell lines, the results demonstrated that circPPP6R3 was slightly downregulated after 24 h while PPP6R3 mRNA was significantly decreased in Caki-1 and ACHN (Fig. 1E). Additionally, circPPP6R3 was resistant to the digestion of RNase R when compared with PPP6R3 mRNA (Fig. 1F), indicating that circPPP6R3 was much more stable than its linear form PPP6R3 mRNA.

### The expression profiles and clinical features of circPPP6R3 in ccRCC

Having confirmed the specific structure of circPPP6R3, we moved forward to detect its expression level in ccRCC cell lines and tissues. Based on the qRT-PCR analysis, circPPP6R3 was significantly highly expressed in four ccRCC cell lines Caki-1, ACHN, 786-O, and Caki-2 relative to HK-2 (Fig. 1G), and we selected Caki-1 and ACHN to continue the downstream regulatory investigation due to their higher expression of circPPP6R3. Next, we employed qRT-PCR to detect the expression of circPPP6R3 in 96 ccRCC tissues and paired normal adjacent renal tissues, and we found that circPPP6R3 was significantly overexpressed in ccRCC tissues (Fig. 1H). Significantly, compared to lower expression of circPPP6R3, higher expression of circPPP6R3 was positively associated with higher histological grade, T stage, and M stage as well as advanced clinical stage of ccRCC tissues (*p* < 0.05, Table 1). Moreover, the Kaplan–Meier analysis indicated that the ccRCC patients with high expression of circPPP6R3 had unfavorable disease-free survival rates compared with low circPPP6R3 expression (*p* < 0.05, Fig. 1I). To summarize, circPPP6R3 was overexpressed both in ccRCC tissues and cell lines, aligned to the online database, and was correlated with malignant progression of ccRCC patients which merits further exploration.

**Table 1:** Correlation between circPPP6R3 expression and clinical features in ccRCC.

Characteristics	Number	No. of patients		*p* value
		High	Low	
Age(y)				0.275
<60	65	30	35	
≥60	31	18	13	
Gender				0.834
Male	59	30	29	
Female	37	18	19	
Histological grade				0.041*
1–2	50	20	30	
3–4	46	28	18	
Clinical stage				0.010*
I–II	77	33	44	
III–IV	19	15	4	
T classification				0.043*
T1-2	82	37	45	
T3-4	14	11	3	
M classification				0.026*
M0	90	42	48	
M1	6	6	0	
N classification				0.432
N0	89	43	46	
N1	7	5	2	

Chi-square test. ^*^p < 0.05; ^**^p < 0.01.

### Knockdown of circPPP6R3 inhibited the proliferation, migration, and invasion of ccRCC cells

**Fig. 2:**
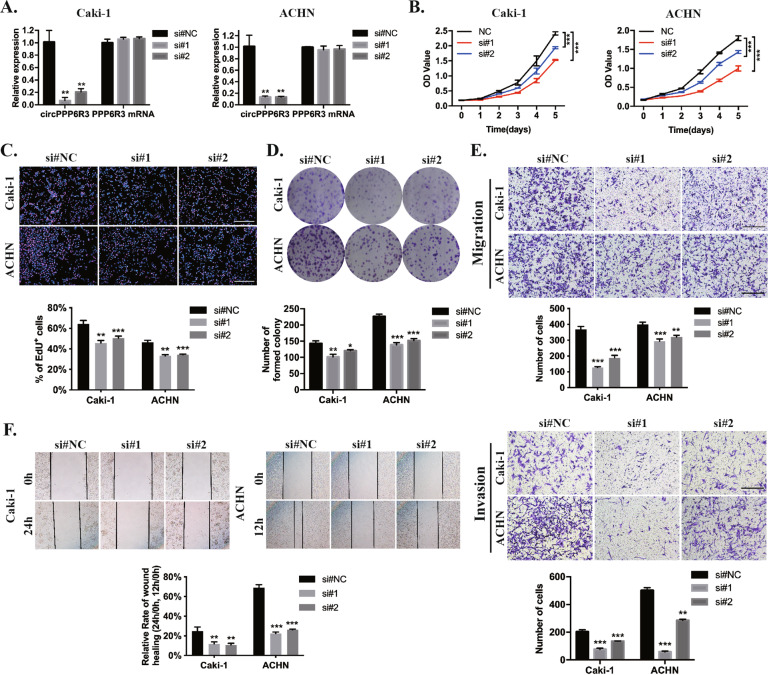
Knockdown of circPPP6R3 suppressed the proliferation, migration, and invasion of ccRCC cells. **A** qRT-PCR detected the expression of circPPP6R3 and PPP6R3 mRNA in Caki-1 and ACHN cells after transfected with siRNAs targeting circPPP6R3. **B**–**D** MTS assay, EdU incorporation assay, and colony formation assay were employed to detect the proliferation of ccRCC cells. **E**, **F** Transwell assay, invasion assay, and wound-healing assay were performed to detect the migrative and invasive abilities of ccRCC cells.

To investigate the biological functions of circPPP6R3, two sequences of siRNAs especially targeting the back-splicing site of circPPP6R3 were synthesized. As the result showed in Fig. 2A, after transfection of the oligonucleotides, the expression of circPPP6R3 was remarkably decreased in Caki-1 and ACHN while no change was observed in the expression of the linear form of PPP6R3 mRNA. As the silencing efficiency of the first siRNAs, si#1, was better than the other one, it was chosen to perform the downstream exploration. MTS assay, EdU corporation assay, and colony formation assay demonstrated that downregulating the expression of circPPP6R3 could significantly inhibit the proliferation ability of ccRCC cells (Fig. 2B–D). Besides, the migratory and invasive abilities were reduced after silencing the expression of circPPP6R3 in ccRCC cells, detected by the transwell assay, invasion assay, and wound-healing assay (Fig. 2E, F).

### Overexpression of circPPP6R3 promoted the tumorigenesis and metastasis of ccRCC cells in vivo

**Fig. 3:**
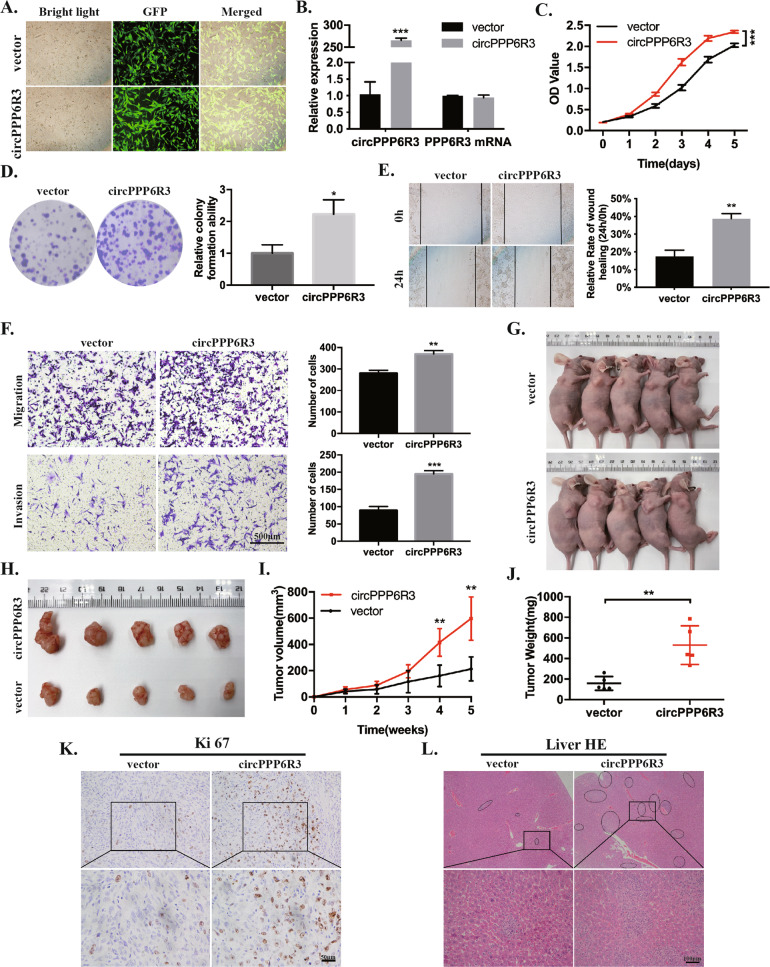
Overexpression of circPPP6R3 promoted the progression of ccRCC. **A** Representative images were shown to exhibit the transfected effects of Caki-1 cells with the vector or circPPP6R3-overexpressing plasmids. **B** The overexpressing efficiency of circPPP6R3 was detected in Caki-1 cells. **C**, **D** MTS assay and colony formation assay were performed to examine the proliferation of Caki-1 cells. **E**, **F** Transwell assay, invasion assay, and wound-healing assay were used to detect the migrative and invasive abilities of Caki-1 cells. **G** The images of the nude mice subcutaneously injected with the vector or circPPP6R3-overexpressed Caki-1 cells were shown. **H** The subcutaneous tumors of stably circPPP6R3-overexpressed or vector group were shown. **I** The growth curves of the subcutaneous tumors were performed by measuring the tumor volumes. **J** The weight of the subcutaneous tumors was measured. **K** Representative IHC images of the subcutaneous tumors stained with Ki67 in circPPP6R3-overexpressed or vector group were shown. **L** Representative HE images of the metastatic tumors in the liver in the hematogenous metastasis model were shown.

To determine the effects of circPPP6R3 in tumorigenesis and metastasis of ccRCC cells in vivo, we first constructed the circPPP6R3-overexpressed or vector control Caki-1 cells by infecting with lentivirus. The transfection efficiency was shown in Fig. 3A, and circPPP6R3 was significantly upregulated in circPPP6R3-overexpressed Caki-1 cells, compared with the vector Caki-1 cells, while the expression PPP6R3 mRNA had no change (Fig. 3B). The capabilities of proliferation, migration, and invasion were elevated in circPPP6R3-overexpressed Caki-1 cells in vitro in comparison with the vector cells (Fig. 3C–F). Then, we subcutaneously injected the circPPP6R3-overexpressed or vector Caki-1 cells in BALB/c nude mice. The tumor volume and the weight of the nude mice were measured every 7 days. Five weeks after injection, the volume of the subcutaneous tumors was much larger in the circPPP6R3-overexpressed group compared to the vector group (Fig. 3G–I), and the tumor weight was much heavier in the circPPP6R3-overexpressed group (Fig. 3J). IHC was subsequently performed to stain Ki67 in the tumor slides, and the results showed that Ki67 was upregulated in the circPPP6R3-overexpressed group in the xenograft tumors, which indicated a stronger capability of growth as well (Fig. 3K). To further investigate the role of circPPP6R3 in metastasis of ccRCC cells in vivo, we injected the circPPP6R3-overexpressed or vector Caki-1 cells into the BALB/c nude mice via their tail veins. After 7 weeks of injection, HE staining of the liver and lung revealed that overexpressing circPPP6R3 could dramatically increase the liver metastatic nodes in nude mice (Fig. 3L). Above all, overexpression of circPPP6R3 promoted the tumorigenesis and metastasis of ccRCC cells in vivo.

### circPPP6R3 served as a sponge for miR-1238-3p

**Fig. 4:**
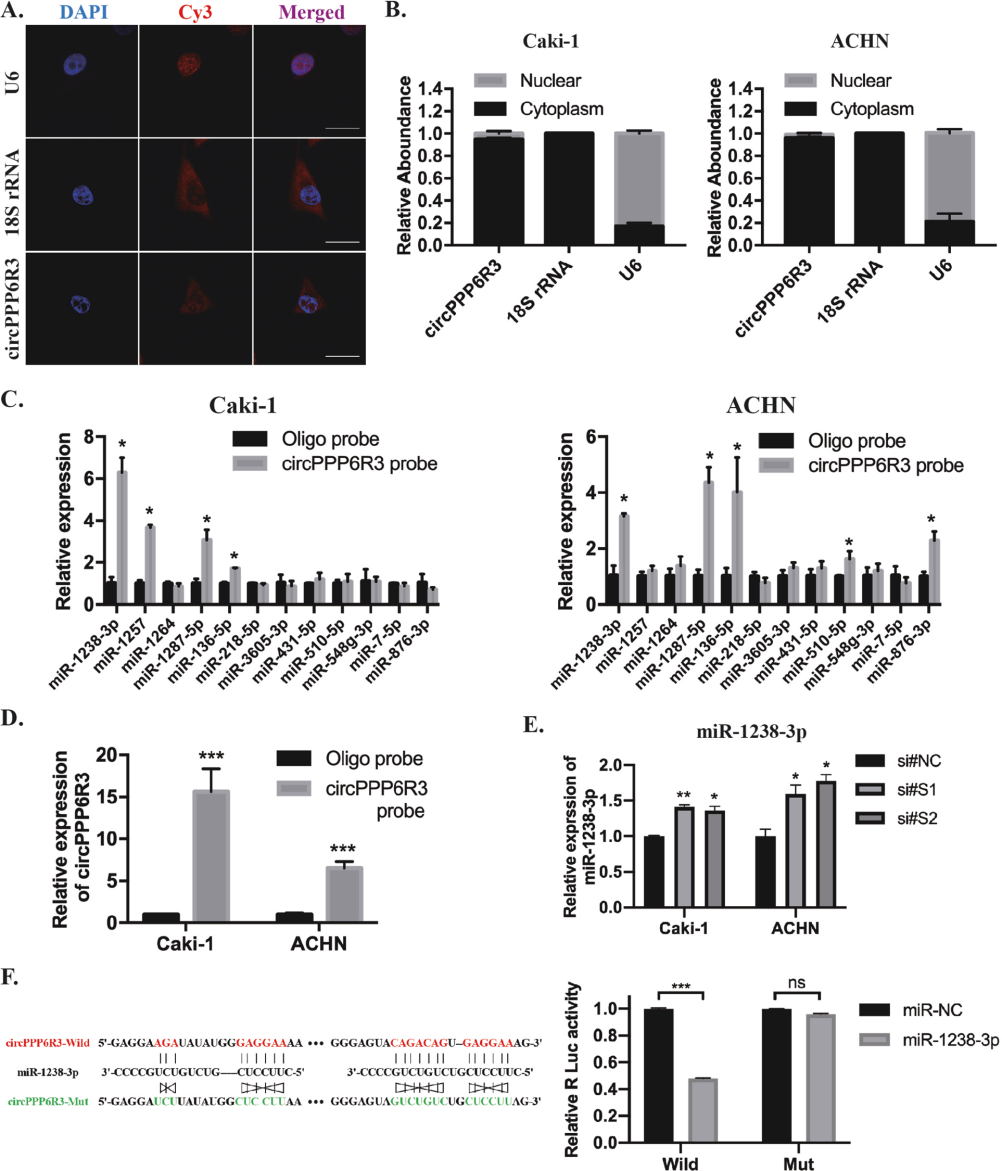
circPPP6R3 served as a sponge for miR-1238-3p. **A** FISH experiment confirmed the localization of circPPP6R3, 18S rRNA, and U6 were used as positive controls respectively for the cytoplasmic and nuclear sublocation. Scale bars, 50 μm. **B** The expression of circPPP6R3 in cytoplasmic and nuclear fractions was validated by qRT-PCR analysis. **C** The miRNAs predicted as the targets of circPPP6R3 were analyzed by qRT-PCR in the biotin-labeled circRNA probe pull-down assay. **D** The biotin-labeled circPPP6R3 probe could capture more circPPP6R3. **E** miR-1238-3p in circPPP6R3-knockdown ccRCC cells was detected by qRT-PCR analysis. **F** Schematic illustration of circPPP6R3 wild type and circPPP6R3 mutant type dual-luciferase reporter vectors, and dual-luciferase reporter assay was performed in the 293T cells to determine the direct binding relationship between circPPP6R3 and miR-1238-3p.

As circRNAs do have several pathways to function in the cells, we first confirmed its cellular sublocation to investigate the mechanisms of circPPP6R3. The nuclear and cytoplasm separation experiment and FISH experiment revealed that circPPP6R3 was predominantly localized within the cytoplasm of ccRCC cells (Fig. 4A, B). Given the fact that circRNAs localized in the cytoplasm preferentially functioned as miRNA sponges, we applied the bioinformatics tool CircInteracgtome, circBank, and Starbase to screen the candidates miRNAs for circPPP6R3. 12 miRNAs, predicted to bind circPPP6R3 by two of the three bioinformatics tools simultaneously, were analyzed in circRNA pull-down assay using a biotin-labeled circPPP6R3 probe or control oligo probe. Among these miRNAs, miR-1238-3p was enriched the most by the circPPP6R3 probe, which could capture more circPPP6R3, compared with the oligo probe in Caki-1 and ACHN cells (Fig. 4C, D). Besides, we detected that the expression of miR-1238-3p was upregulated in circPPP6R3-knockdown ccRCC cells (Fig. 4E). Subsequently, to evaluate the binding relationship between circPPP6R3 and miR-1238-3p, we constructed the wild-type dual-luciferase reporter vector of circPPP6R3. The results showed that miR-1238-3p could significantly decrease the Renilla luciferase activity compared to the miR-NC group by dual-luciferase reporter assay. However, when we mutated the complementary binding sites of miR-1238-3p in circPPP6R3 carried psiCHECK-2, we found no change of Renilla luciferase activity with the transfection of miR-1238-3p compared to miR-NC (Fig. 4F). Collectively, these results indicated that circPPP6R3 might function as a sponge for miR-1238-3p in ccRCC cells.

### circPPP6R3 functions by interacting with miR-1238-3p in ccRCC cells

**Fig. 5:**
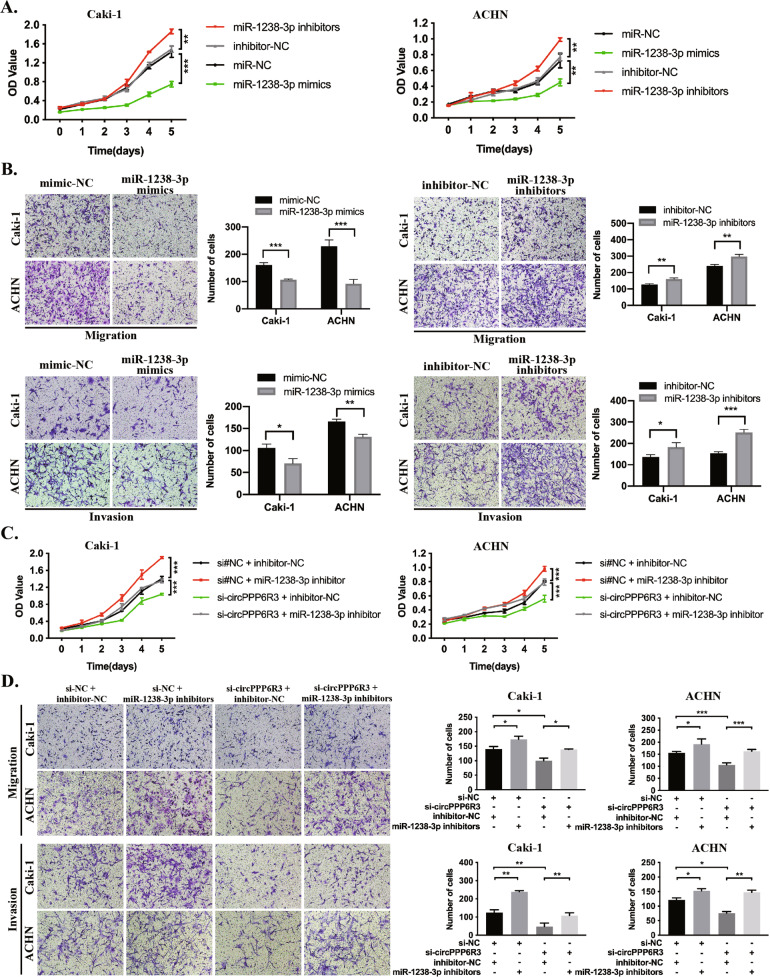
circPPP6R3 functions through interacting with miR-1238-3p in ccRCC cells. **A** The MTS assay was conducted to evaluate the ability of proliferation of miR-1238-3p in ccRCC cells. **B** The migration and invasion assay were employed to verify the role of miR-1238-3p on metastatic capacity in ccRCC cells. **C** The MTS assay was adopted to investigate the ability of proliferation of miR-1238-3p inhibitors in circPPP6R3-knockdown ccRCC cells. **D** The migration and invasion assay was conducted to validate the metastatic function of miR-1238-3p inhibitors in circPPP6R3-knockdown ccRCC cells.

miR-1238-3p was once reported to be a repressor in cutaneous squamous cell carcinoma [23], but none of the biological functions was known in ccRCC. Therefore, we began our investigation by transfecting the miR-1238-3p mimics or inhibitors into the ccRCC cells. Functionally, miR-1238-3p mimics could significantly repress the proliferation, migration, and invasion capacities in ccRCC cells. In contrast, miR-1238-3p inhibitors could remarkably achieve the reverse effects (Fig. 5A, B). To further demonstrate whether circPPP6R3 exerts its regulatory effect via sponging miR-1238-3p in ccRCC, we conducted a rescue experiment to verify the functional interaction between circPPP6R3 and miR-1238-3p. We first co-transfected inhibitor-NC/miR-1238-3p inhibitors and si#NC/si# circPPP6R3 into ccRCC cells. The cell proliferation assay confirmed that circPPP6R3-knockdown-induced suppression of cellular proliferation could be attenuated by miR-1238-3p inhibitors (Fig. 5C). Besides, the migration and invasion assay revealed that miR-1238-3p inhibitors had a rescue effect on the repression of migratory and invasive capacities by knocking down circPPP6R3 (Fig. 5D). To sum up, these results suggested that circPPP6R3 exerts its biological function through interacting with miR-1238-3p in ccRCC cells.

### circPPP6R3 upregulated CD44 via sponging to miR-1238-3p

**Fig. 6:**
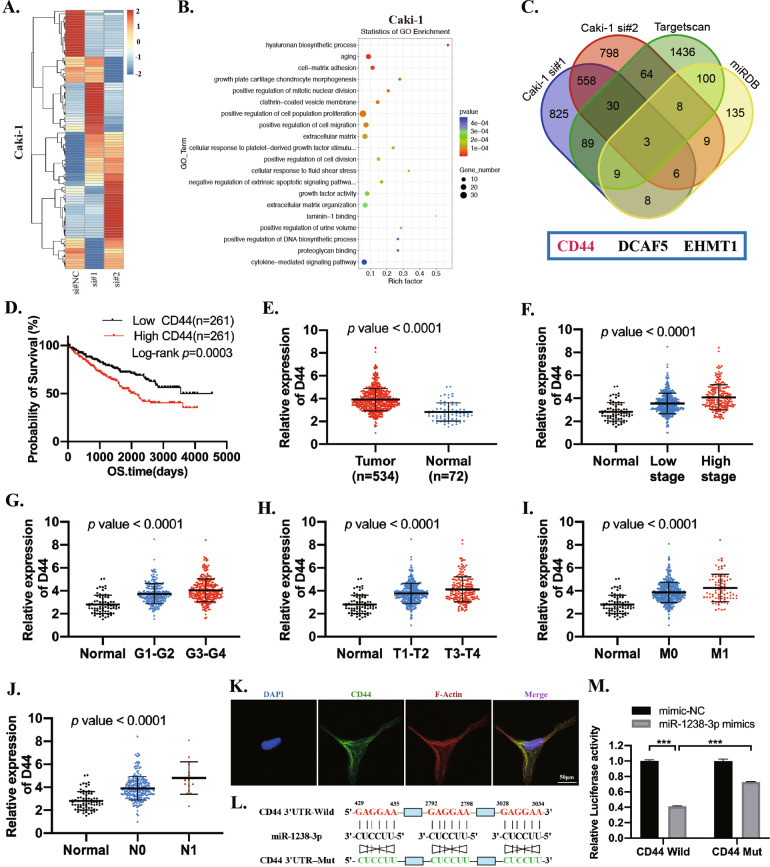
The identification and clinical characteristics of CD44 in ccRCC. **A** Heatmap for the differentially expressed genes in circPPP6R3-knockdown Caki-1 cells. **B** GO analysis was performed for the differentially expressed genes. **C** Venn diagram was performed to screen the potential targets of miR-1238-3p by MiRPathDB, Targetscan and RNA-seq. **D** Kaplan–Meier analysis indicated that high expression of CD44 predicted poor survival probability. **E** Expression of CD44 in ccRCC based on TCGA samples. **F**–**J** Relative expression of CD44 in ccRCC with different clinical stages, pathological grades, T stages, M stages, and N stages from TCGA. **K** Representative IF images of ACHN cells stained with CD44 were shown. **L** Sequence of CD44 3'UTR binding to miR-1238-3p, predicted by Targetscan, and relative mutated sequence were shown. (Totally three predicted binding sites were mutated). **M** Dual-luciferase reporter assay was performed in 293T cells to determine the interacting relationship between miR-1238-3p and CD44.

To investigate the downstream regulations of circPPP6R3, we performed RNA-sequencing to verify the target genes (Fig. 6A). Totally, 651 genes were downregulated simultaneously in siRNA#1 and siRNA#2 targeting circPPP6R3 in Caki-1 cells (fold change >2, *p* < 0.05, Supplementary File 2). Gene Ontology Enrichment analysis revealed that these differentially expressed genes involved in cell-matrix adhesion, positive regulation of cell migration as well as positive regulation of cell population proliferation (Fig. 6B), which was consistent with the biological changes. It is well known that miRNAs could bind to the 3'UTR of the mRNA to regulate the expression of their target genes [24]. Thus, we employed miRDB and Targetscan to identify the potential target genes of miR-1238-3p. By taking the intersection of the predicted target genes with the genes downregulated in circPPP6R3-knockdown Caki-1 cells, 3 potential genes stood out and were selected for further investigation (Fig. 6C). Among these candidate genes, CD44 was the only one that was correlated with the unfavorable prognosis of ccRCC patients (Fig. 6D). Additionally, CD44 was upregulated in ccRCC tissues (*n* = 533) compared with normal renal tissues (*n* = 72) in TCGA samples (Fig. 6E and Supplementary File 3), and its expression levels were positively associated with the clinical stage, pathological stage, T stage, N stage, and M stage of ccRCC patients (Fig. 6F–J). CD44 is a cell-surface glycoprotein involved in cell-cell interactions, cell adhesion, and migration [25] (Fig. 6K). To further confirm whether CD44 actually interacted with miR-1238-3p, we conducted the dual-luciferase reporter assay by co-transfecting CD44 3’UTR-Wild or CD44 3’UTR-Mut with miR-1238-3p mimics or mimic-NC in 293 T cells. As shown in Fig. 6L, M, miR-1238-3p mimics could significantly reduce the Renilla luciferase activity in CD44 3’UTR-Wild transfecting cells. Furthermore, the Renilla luciferase activity could be rescued to a large extent when we mutated the miR-1238-3p binding sites in plasmids containing CD44 3'UTR, indicating an interacting relationship between miR-1238-3p and CD44.

**Fig. 7:**
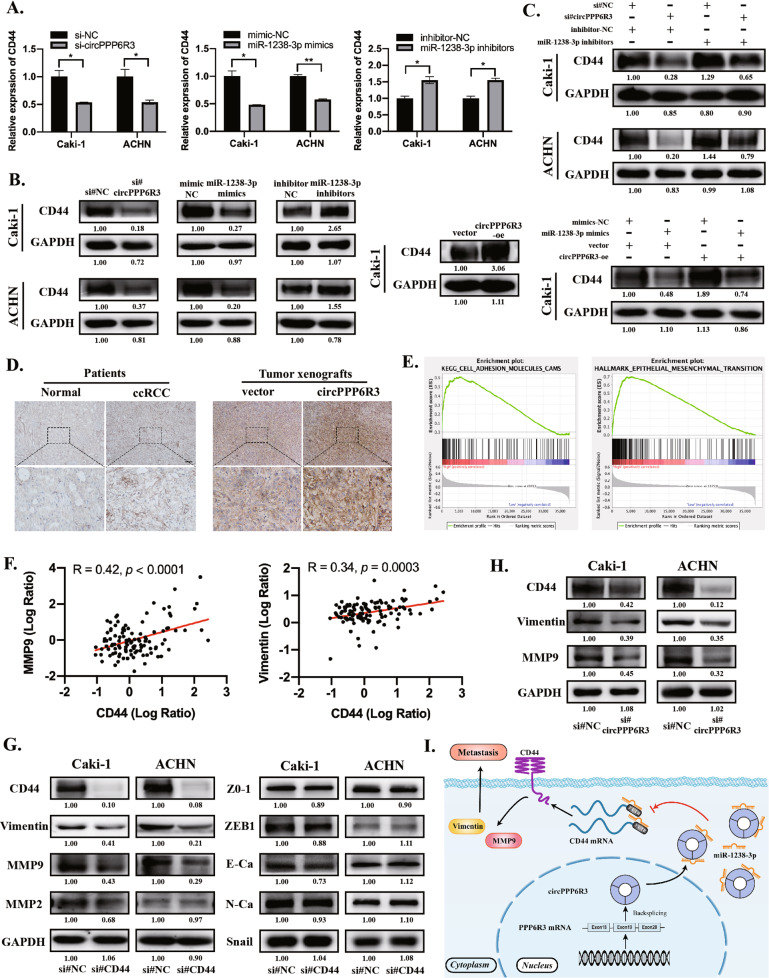
circPPP6R3 upregulated CD44 via sponging to miR-1238-3p. **A**, **B** qRT-PCR and Western Blotting were conducted to detect the relative expression of CD44 in ccRCC cells treated with siRNAs and overexpressing plasmids for circPPP6R3, mimics, and inhibitors for miR-1238-3p. **C** The circPPP6R3-knockdown-induced diminishment of CD44 was attenuated by miR-1238-3p inhibitors in ccRCC cells. **D** Representative IHC images of CD44 in ccRCC tissues as well as xenografts tumor tissues in animal studies. Scale bars, 50 μm. **E** GSEA was performed to identify the downstream pathway of CD44 in TCGA patients based on the high and low expression of CD44. **F** Correlation analysis of the protein level between CD44 and MMP9, Vimentin from the database in CPTAC. **G**, **H** MMP9 and Vimentin were downregulated in CD44-silencing or circPPP6R3-silencing ccRCC cells. **I** Schematic diagram illustrating the biological functions and mechanisms of the regulatory axis of circPPP6R3/miR-1238-3p/CD44 in ccRCC progression.

Next, we verified that CD44 was downregulated when transfected with siRNAs for circPPP6R3 or miR-1238-3p mimics both in RNA and protein levels in ccRCC cells, while an upregulation effect was observed when transfected with circPPP6R3-overexpressing plasmids or miR-1238-3p inhibitors (Fig. 7A, B). Furthermore, we demonstrated that the circPPP6R3-knockdown-induced diminishment of CD44 could be attenuated by miR-1238-3p inhibitors in Caki-1 and ACHN cells (Fig. 7C). We also confirmed that CD44 was highly expressed in our collected ccRCC tissues and its expression level was augmented with the overexpression of circPPP6R3 in our animal studies, representative images of IHC were shown (Fig. 7D). Next, to analyze the downstream pathway of CD44, we performed Gene Set Enrichment Analysis (GSEA) and noticed that the hallmarks of epithelial-mesenchymal transition (EMT) and the pathway of cell adhesion molecules were involved (Fig. 7E). With the employment of the Clinical Proteomic Tumor Analysis Consortium (CPTAC), the protein level of MMP9 and Vimentin were positively associated with CD44 in ccRCC tissues (Fig. 7F). Therefore, we constructed siRNAs targeting CD44 to detect the relevant molecules involved in the EMT pathway. The results showed that the expression of MMP9 and Vimentin were indeed reduced along with the knockdown of CD44 in ccRCC cells, leading to a restraint of EMT (Fig. 7G). Additionally, MMP9 and Vimentin were also diminished in circPPP6R3-silencing ccRCC cells (Fig. 7H). Taken together, these results implied that circPPP6R3 could promote the progression of ccRCC via the miR-1238-3p/CD44 axis (Fig. 7I).

## Discussion

circRNAs, first discovered as viroids in 1976 [26], were formed by precursor mRNA back-splicing or skipping events of genes in eukaryotes [27]. Along with the applications of high-throughput RNA-sequencing and circRNA specific bioinformatics algorithms, an increasing number of circRNAs were confirmed to be related to various diseases, especially in human malignancies like bladder cancer [28], breast cancer [29], gastric cancer [30], and so forth. Nevertheless, to date, the pivotal roles of circRNAs in the proliferation and metastasis of RCC are still not well-known and warrant further exploration. In this study, a novel oncogenic circRNA derived from PPP6R3, circPPP6R3, was remarkably upregulated both in ccRCC tissues and cell lines through co-analyzing the data from GSE100186 with the Caner Specific circRNA network database. circPPP6R3 was positively associated with pathological grade, TNM stage, tumor size, and vascular invasion of ccRCC. Further investigations revealed that circPPP6R3 could promote the proliferation and metastasis of ccRCC in vitro and in vivo, indicating a potential application of circPPP6R3 in the diagnosis and prognosis of ccRCC. Moreover, we first report that circPPP6R3 promoted ccRCC progression via competitively binding miR-1238-3p and upregulated CD44.

Numerous studies have shown that acting as a miRNA sponge is an important way for cytoplasmic localized circRNAs to function in tumor cells, which means these circRNAs could competitively bind with specific miRNAs to antagonize the miRNA-mediated post-transcription regulation. circPPP6R3 was back-spliced of the exon 18,19 and 20 originated from the PPP6R3 gene and was predominantly localized within the cytoplasm of ccRCC cells, indicating its potential to function through binding with miRNAs. Therefore, we performed bioinformatic analysis and found that circPPP6R3 possessed two miR-1238-3p binding sites and could directly bind to miR-1238-3p with the adoption of circRNA pull-down assay as well as dual-luciferase reporter assay. miR-1238-3p was reported to be a tumor suppressor in non-small cell lung cancer [31] and cutaneous squamous cell carcinoma [23], but its biological function in ccRCC has not been investigated so far. Our exploration determined that miR-1238-3p played a repressive role in the proliferation, migration, and invasion of ccRCC cells. Further rescue experiments confirmed that miR-1238-3p inhibitors could attenuate the circPPP6R3-knockdown-induced suppression of proliferative and metastatic capacities of ccRCC cells, supporting the conjecture that circPPP6R3 functions via acting as a sponge for miR-1238-3p.

It has been demonstrated that miRNAs play significant roles in cancer progression through post-transcriptional regulation of their target genes. To identify the target genes of miR-1238-3p, bioinformatic analysis and RNA-sequencing were employed and the results indicated that CD44 was potentially the target of miR-1238-3p, with 3 binding sites in the 3'UTR of CD44 mRNA. Aberrantly upregulated in multiple carcinomas, like breast cancer [32], lung adenocarcinoma [33], colorectal cancer [34], prostate cancer [35], and RCC [36], CD44 takes part in various physiological processes including tumorigenesis and cancer progression [37]. Previous studies revealed that CD44 plays a pivotal role in regulating the diverse process of RCC pathogenesis [36, 38]. Herein, we confirmed an upregulated expression of CD44 in ccRCC tissues and its positive correlation with the clinical stage, pathological stage, T stage, N stage, M stage, and poor prognosis of ccRCC patients. The interacting relationship between CD44 and miR-1238-3p was verified by the dual-luciferase reporter assay. In addition, we determined that the expression level of CD44 could be regulated by circPPP6R3 and miR-1238-3p. CD44 has been reported to be involved in the regulation of diverse molecules and pathways, such as EMT-related molecules, MMPs [39], cycle-related proteins [40], glycolysis-related genes [41], Wnt/β-catenin pathway [40], and PI3K-Akt pathway [42], which modulated the proliferation, metastasis, and therapy-resistance of cancer cells. Moreover, we analyzed the downstream of CD44 in ccRCC through GSEA analysis and focused on EMT and cell adhesion-related molecules. Further investigation revealed that MMP9 and Vimentin were regulated by CD44 in ccRCC. Thus, we speculated that a modulatory network of circPPP6R3/miR-1238-3p/CD44 was involved in the progression of ccRCC.

## Conclusions

In conclusion, our investigations demonstrated that circPPP6R3 was highly expressed in ccRCC tissues and was associated with inferior clinical characteristics of ccRCC patients. Mechanism analysis revealed that circPPP6R3 could serve as a sponge for miR-1238-3p to upregulate the expression of CD44, which modulated the proliferation, migration, and invasion of ccRCC. The regulatory network involving circPPP6R3/miR-1238-3p/CD44 axis provided an insight into understanding the development and progression of ccRCC, and might offer a promising biomarker as well as a therapeutic approach for ccRCC.

## Materials and methods

### Patient tissue specimens

Totally, 96 paired ccRCC tissues and matched normal renal tissues were collected from patients who underwent radical or partial nephrectomy at Sun Yat-sen Memorial Hospital, Sun Yat-sen University between 2016 and 2020. The histological characteristics of specimens were confirmed by pathologists in accordance with the 2016 World Health Organization Consensus Classification and Staging System for RCC as well as Fuhrman grade. All procedures were conducted with the approval of the Ethics Committee of Sun Yat-sen Memorial Hospital, Sun Yat-sen University. All patients had already signed the written informed consent.

### Fluorescence in situ hybridization (FISH)

Cy3-labeled circPPP6R3 probe was purchased from GenePharma (China). ccRCC cells were seeded in a 15 mm confocal dish and were conducted with Fluorescence in situ hybridization kit GenePharma (China). Briefly, Caki-1 cells with the fixation of 4% paraformaldehyde were incubated with Cy3-labeled circPPP6R3 probe overnight, stained with DAPI and finally photographed by ZEISS LSM800 confocal microscope (Germany).

### Biotin-labeled probe pull-down assay

The Biotin-labeled circPPP6R3 probe synthesized from GenePharma (China) was especially complemented to the back-spliced junction of circPPP6R3. Approximately 1 × 10^7^ ccRCC cells were harvested, fixed by 1% paraformaldehyde and lysed with 100 μl lysis buffer. Then, the cells were incubated with the probe-attached streptavidin dynabeads (M-280, Invitrogen, USA) at 4 °C overnight. The next day, the dynabead-probe-circPPP6R3 mixture was washed, incubated with 200 μl lysis buffer supplemented with proteinase K at 25 °C to loosen the formaldehyde cross-linking. Then, total RNA was isolated with Trizol reagent to detect the expression of circPPP6R3 and relative miRNAs by qRT-PCR.

### RNA-sequencing and bioinformatics analysis

RNA-sequencing was performed to identify the genes regulated in circPPP6R3-knockdown Caki-1 cells relative to the negative control cells. A criterion of fold change >2 and *p* < 0.05 was adopted to screen the differentially expressed genes which would be selected for further analysis.

CircInteracgtome [43], circBank [44], and starBase [45] were employed to screen the candidates miRNAs for circPPP6R3. miRDB [46] and Targetscan [47] were adopted to analyze the potential target genes of miR-1238-3p. GSEA [48] was performed to verify the downstream pathway of CD44. CPTAC was employed to analyze the correlation between MMP9, Vimentin, and CD44.

### Dual-luciferase reporter assay

The wild-type and mutant reporter plasmids (psicheck2-Firefly Luciferase-Renilla Luciferase) were purchased from IGE Biotech Co (China). The Renilla Luciferase activity and the Firefly Luciferase activity were measured 48 h later after the transient transfection of both reporter plasmids and miRNA mimics in accordance with the manufacture’s protocols (E292, Promega, USA). The Renilla Luciferase activity was normalized to the Firefly Luciferase activity when comparing the wild type and mutant group.

### In vivo experiments

To further determine the proliferative function of circPPP6R3, ten 6-week BALB/c female nude mice were chosen for tumor xenografts models and divided into two groups (*n* = 5). In total, 150 μl 3 × 10^6^ stably circPPP6R3-overexpressing or vector Caki-1 cells were injected into the right upper back of the mice subcutaneously. The weight of the nude mice, as well as the width and length of the tumor, was measured every 7 days with a caliper, the volume of the tumor was calculated as (length × width^2^)/2. After 5 weeks, the nude mice were sacrificed, tumors were excised for further evaluation including the weight, circPPP6R3 expression, pathological examination and molecules testing. For investigating the role of circPPP6R3 on hematogenous metastasis, 125 μl 2.5 × 10^6^ stably circPPP6R3-overexpressing or vector Caki-1 cells were injected into the tail vein of the nude mice. Seven weeks later, the livers and lungs were resected after the euthanasia, embedded in paraffin and followed by HE staining.

### Statistical analysis

All data were presented as means ± standard error of the mean carried out by SPSS 20 software or GraphPad Prism 8.0. The Chi-square test or Fisher’s exact test was performed to analyze the correlation between the expression level of circPPP6R3 and clinical-pathological characteristics. The Student’s *t* test was employed to evaluate the significant difference between the two independent groups. Kaplan–Meier analysis with log-rank test was used to compare the disease-free survival and overall survival rates. *p* < 0.05 was considered statistically significant. *p < 0.05, **p < 0.01, ***p < 0.001.

## Supplementary information

Supplementary File 1

Supplementary File 2

Supplementary File 3

Supplementary File 4

## Data Availability

The RNA-seq data of Caki-1 cells treated with siRNAs for circPPP6R3 analyzed in this study are included in Supplementary File 2. The rest datasets used or analyzed during the current study are available from the corresponding author on reasonable request.
