# Supplementary material for: Circular RNA circPPP6R3 upregulates CD44 to promote the progression of clear cell renal cell carcinoma via sponging miR-1238-3p
Source: Cell Death Dis. 2021 Dec 21;13(1):22. doi: 10.1038/s41419-021-04462-5 (PMC8692348; doi:10.1038/s41419-021-04462-5)
Supplement: Supplementary file 4 [file 41419_2021_4462_MOESM4_ESM.pdf]

Supplementary material and methods

Cell lines and cell culture

The ccRCC cell lines Caki-1, ACHN, 786-O and HK-2, the normal kidney tubular epithelial cell line, were purchased from American Type Culture Collection. Caki-1 cells were cultured in McCoy's 5A Modified medium (Gibco, USA); 786-O and ACHN cells were cultured in RPMI-1640 medium (Gibco, USA); HK-2 cells were cultured in DMEM/F-12 medium (Gibco, USA); 293T cells were cultured in DMEM medium (Gibco, USA), supplemented with 10% FBS (Ruiyan Bio, Uruguay) and 1% penicillin/streptomycin (Gibco, USA) at 37 ℃ supplied with 5% CO_2 in a humidified atmosphere.

RNA extraction, RNase R treatment, and quantitative real-time PCR assays

The isolation of total RNA from tissues and cell lines was extracted with Trizol reagent (TaKaRa, Japan). For the RNase R treatment, an amount of 2 mg total RNA was incubated with or without 3 U/mg RNase R (Epicentre Technologies, USA) at 37° C for 15 min. To investigate the expression of mRNA and circRNA, 500 μg of total RNA was subjected to synthesize complementary DNA using the reverse transcription kit PrimeScript RT Master Mix (TaKaRa, Japan). For miRNA analysis, Mir-X^{TM} miRNA First-Strand Synthesis kit (TaKaRa, Japan) was used. The qRT-PCR was conducted with the SYBR Premix Ex Taq^{TM} kit (TaKaRa, Japan) using LightCycler 480 instrument (Roche Diagnostics). GAPDH acted as an internal standard control for mRNA and circRNA analysis while U6 for miRNA analysis using the $2^{-\Delta\Delta CT}$ method.

Nucleic acid electrophoresis

To confirm the products amplified by the primers of circPPP6R3 were circular form, gDNA and cDNA PCR products were separated in 2% agarose gel with TAE running buffer using electrophoresis system at 120V for 30 min, followed by UV irradiation. Super DNA Marker (CWBIO, China) was applied as a DNA marker and GAPDH acted as a control.

Small interference RNAs (siRNAs), plasmids construction and stable transfection

siRNAs targeting circPPP6R3 were obtained from RiboBio (China) and were transfected into ccRCC cell lines with Lipofectamine iMax (Invitrogen, USA). The miRNA mimics, inhibitors, and respective control were synthesized by GenePharma (China). The circPPP6R3-overexpressing plasmids were synthesized by IGE Biotech Co (China). Plasmids were co-transfected with lentiviral packaging plasmids into 293T cells to package lentivirus. And then, Caki-1 cells were infected by lentivirus to stably overexpressing circPPP6R3 followed by 7-day selecting with 2ug/mL puromycin.

Cell proliferation assay

For the MTS assay to detect the cell viability, 1000 transfected cells were seeded and cell viability was detected with MTS reagents (Promega, USA) for 6 days. An Absorbance Microplate Reader (CMAX PLUS, China) was utilized to read the absorbance at a wavelength of 490 nm.
For colony formation assay, 1000 transfected cells were respectively seeded into 6-well plates and were cultured for 7-10 days. Afterward, the colonies were fixed with 4% paraformaldehyde and were stained with 0.1% crystal violet, counted and photographed.

Transwell assay and invasion assay

For transwell assays, 200 ul 10^5 transfected ccRCC cells were plated on the upper chamber of transwell chamber inserts (Corning, NY) with or without precoated Matrigel (Corning, NY) as instructed by the manufacturer's protocol. Then, the lower chambers were filled with 600 μl complete medium. After a period of incubating, the migratory or invasive cells were fixed with 4% paraformaldehyde, dyed with crystal violet and were counted in three randomly selected fields with a Nikon Eclipse 80i system (Nikon, Japan).

Wound Healing Assay

The transfected ccRCC cells were seeded in a 12-well culture plate and cultured in a humidified atmosphere overnight. The next day, the monolayer cells were scratched with a 200 ul pipette tip when reached 90% confluence and were photographed with a Nikon Eclipse 80i system. Then, the cells were washed with PBS gently and cultured with FBS-free medium at 37 °C and 5% CO_2. The cell migration was photographed at the same sight 12 h or 24 h later.

5-Ethynyl-20-deoxyuridine (EdU) incorporation assay

The EdU incorporation assay was performed to validate the cellular proliferative status with a EdU-related Kit (RiboBio, China). 10^4 ccRCC cells were plated in 96-wells in advance and then cultured with EdU for 2 h. These cells were fixed using 4% paraformaldehyde and were incubated with Apollo Dye Solution and Hoechst 33342 in order according to the manufacturer's instructions. The ratio of EdU positive cells to Hoechst-stained cells was calculated to evaluate the cell proliferation after photographing and counting under an Olympus IX73 microscope(Olympus, Japan).

Cell immunofluorescence (IF) staining

Briefly, ACHN cells seeded in the confocal dish were fixed with 4% formaldehyde solution for 15 min, permeated with 0.1% Triton X-100 for 20 min, blocked by 10% goat serum for 2 h, and then stained with primary antibodies anti-CD44 (15675-1, Proteintech Group, China) and Phalloidin (to stain F-actin, A12380, Invitrogen, USA) overnight at 4 °C. The next day, cells were incubated with Alexa Fluor 488 (R37116, Invitrogen, USA) at 37 °C for 1 h, followed by DAPI (4',6-diamidino-2-phenylindole, D8200, Solarbio, China) staining for 5 min, and then photographed by ZEISS LSM800 confocal microscope (Carl Zeiss AG, Germany).

Immunohistochemistry (IHC).
Immunohistochemistry was performed according to the protocol of Biotin-Streptavidin HRP Detection Systems (ASGB-BIO, China). Briefly, tissues were first fixed in 4% formaldehyde, embedded in paraffin, and then cut into 5-μm sections. Next, primary antibody anti-CD44 (1:250, 15675-1, Proteintech Group, China) or anti-Ki-67 (ZM-0165, ASGB-BIO, China) were used to incubate the sections for overnight at 4°C. A Nikon Eclipse 80i system (Nikon, Japan) was adopted to capture the images and three random sites of each slide were detected. The expression of target proteins was detected by the assessment of the proportions and intensities of positive cells.

Western Blotting analysis

ccRCC cells or tissues were lysed in RIPA (CWBIO, China) combined with phosphatase and protease inhibitors (Roche, Switzerland) to extract protein. The quantities of the protein were detected with the BCA Protein assay kit (CWBIO, China). Then, an equal quantity of protein was electrophoresed by SDS-PAGE gels at 120V, transferred onto PVDF membranes which would be blocked by 5% BSA and

incubated with specific primary antibodies at 4°C overnight. The next day, membranes were washed by TBST, incubated with HRP-conjugated secondary antibodies and washed three times with TBST before forming an image by G:BOX Chemi XT4 (SYNGENE, USA) with Immobilon ECL substrate (Millipore, Germany).

Primers, siRNAs and probes

Primers	Sequence
circPPP6R3-F	CCCAACTGGTCAGCTAACTT
circPPP6R3-R	ACAAGGCAATATTTCCCTCAGG
PPP6R3-F	CATGGGACACCTTACGAGGAT
PPP6R3-R	GTGACTCACTTGCTGTAAAGAGG
GAPDH-F	CAATGACCCCTTCATTGACC
GAPDH-R	TTGATTTTGGAGGGATCTCG
divergent GAPDH-F	GAAGGTGAAGGTCGAGTC
divergent GAPDH-R	GAAGATGGTGATGGGATTTC
CD44-F	CTGCCGCTTTGCAGGTGTA
CD44-R	CATTGTGGGCAAGGTGCTATT
18S rRNA-F	ACACGGACAGGATTGACAGA
18S rRNA-R	GGACATCTAAGGGCATCACA
U6	Clontech
miR reverse	Clontech
miR-1238-3p	TATACTTCCTCGTCTGTCTGCCCC
miR-1257	CGCAGTGAATGATGGGTTCTGACC
miR-1264	GCGCAAGTCTTATTTGAGCACCTGTT
miR-1287-5p	TGCTGGATCAGTGGTTCGAGTC
miR-136-5p	CGCGACTCCATTTGTTTTGATGATGGA
miR-218-5p	CGCGTTGTGCTTGATCTAACCATGT
miR-3605-3p	CCCTCCGTGTTACCTGTCCTCTA
miR-431-5p	TATGTCTTGCAGGCCGTCATGC
miR-510-5p	CGTACTCAGGAGAGTGGCAATCAC
miR-548g-3p	CGCGCGCGAAAACTGTAATTACTTTTG
miR-7-5p	CGCGCGTGGAAGACTAGTGATTTTG
miR-876-3p	CGCGCTGGTGGTTTACAAAGTAATTCA
**siRNAs**	**Sequence**

si#NC	RiboBio supplied
si-circPPP6R3#1	CTTCCCTGAGGGAAATATT (Target Sequence)
si-circPPP6R3#2	CCTGAGGGAAATATTGCCT (Target Sequence)
si-CD44#1	sense : 5’- GCGCAGAUCGAUUUGAAUATT -3’<br>antisense: 5’- UAUUCAAAUCGAUCUGCGCTT -3’
**miRNA mimics and inhibitors**	**Sequence**
mimics NC	sense : 5’- UUCUCCGAACGUGUCACGUTT -3’<br>antisense: 5’- AUGUGACACGUUCGGAGAATT -3’
inhibitors NC	5’- CAGUACUUUUGUGUAGUACAA -3’
miR-1238-3p mimics	sense : 5’- CUUCCUCGUCUGUCUGCCCC -3’<br>antisense: 5’-GGCAGACAGACGAGGAAGUU -3’
miR-1238-3p inhibitors	5’- GGGGCAGACAGACGAGGAAG -3’
**FISH probes**	**Sequence**
Cy3- circPPP6R3	5’-CAAACAAGGCAATATTTCCCTCAGGGAAGACGTGAACT-3’
Cy3- U6	5’- TTTGCGTGTCATCCTTGCG -3’
Cy3- 18S rRNA	5’- CTTCCTTGGATGTGGTAGCCGTTTC -3’
**circRNA pulldown probes**	**Sequence**
Biotin- circPPP6R3 probes	5’-CAAGGCAAUAUUUCCCUCAGGGAAGACGUGAACUCTG-3’
Biotin- NC probes	5’- UUGUACUACACAAAAGUACUG -3’
